# Copepod-Associated Gammaproteobacterial Alkaline Phosphatases in the North Atlantic Subtropical Gyre

**DOI:** 10.3389/fmicb.2020.01033

**Published:** 2020-05-25

**Authors:** Katyanne M. Shoemaker, Elizabeth A. McCliment, Pia H. Moisander

**Affiliations:** Department of Biology, University of Massachusetts Dartmouth, North Dartmouth, MA, United States

**Keywords:** metatranscriptome, copepod microbiome, zoosphere, alkaline phophatase, gammaproteobacteria, *Vibrio*, copepod associates

## Abstract

Planktonic organisms may provide a niche to associated bacteria in the oligotrophic ocean. Bacterial fitness strategies in association with copepods – abundant planktonic crustaceans – were examined by sampling and incubation experiments in the North Atlantic Subtropical Gyre (NASG). The bacterial metatranscriptome was dominated by Gammaproteobacteria and showed expression of complete bacterial pathways including chemotaxis, cell signaling, and alkaline phosphatase activity. Quantitative PCR and reverse transcriptase qPCR revealed the consistent presence and expression of alkaline phosphatase genes primarily by *Vibrio* spp. in the copepod association. Copepod-associated bacteria appear to respond to prevailing phosphorus limitation by using alkaline phosphatases to break down organophosphoesters, presumably originating from the copepods. The results suggest that the basin-wide tendency for phosphorus limitation in the North Atlantic Ocean is occurring at microscales in these nitrogen-enriched copepod microenvironments. The bacterial communities and their fitness strategies supported by associations with these abundant mesozooplankton are unique from the surrounding seawater and could have large-scale implications for biogeochemical cycling, marine food web structuring, and copepod and ecosystem health.

## Introduction

Growth of free-living plankton in the oligotrophic ocean can be limited or co-limited by the availability of various elements including organic carbon, nitrogen (N), iron, or phosphorus (P) (Ryther and Dunstan, 1971; Falkowski, 1997; Cavender-Bares et al., 2001; Moore et al., 2008, 2013). Soluble reactive phosphorus (SRP) levels in the North Atlantic Ocean stratified surface layers generally remain low and overall, North Atlantic is considered a relatively P limited oceanic basin. The SRP concentrations are an order of magnitude lower in the oligotrophic surface waters of the Bermuda Atlantic Time Series study site (BATS) than at the Hawaii Ocean Time Series Station (station ALOHA) in the North Pacific Subtropical Gyre (Cavender-Bares et al., 2001). The SRP pool has a turnover time of only 3–13 h at BATS compared to the 9-day turnover time estimated at station ALOHA (Ammerman et al., 2003). Plankton communities are therefore more likely to express P limitation in the North Atlantic.

N and P are considered co-limiting nutrients for bacterial growth in the North Atlantic, and bacteria attached to living organisms may benefit from nutrients originating from the host. Copepods, an abundant component of the mesozooplankton, release substantial amounts of ammonium into the surrounding seawater, which may be assimilated by nearby bacteria (Verity, 1985; Saba et al., 2011). Dissimilatory nitrate reduction in copepod association is also active, suggesting sufficient DIN is available for respiration (Moisander et al., 2018). Therefore, bacteria associated with copepods in the North Atlantic may not face N limitation to the extent of the surrounding free-living community.

We conducted metatranscriptomic sequencing followed by quantitative PCR (qPCR) and reverse transcriptase qPCR (RT-qPCR) analyses to examine nutrient acquisition and other fitness strategies of bacteria associated with copepods. P limitation in the microbial community would be expected to be detected as gene expression of bacterial pathways involved with use of dissolved organic P (DOP) compounds. While SRP availability is low in NASG surface waters, a larger pool of DOP is available year-round. The pool of DOP is composed mostly of phosphonates (C-P bond) and phosphoesters (C-O-P bond), in a 1:3 ratio, regardless of depth in the North Pacific (Clark et al., 1998; Kolowith et al., 2001). Many bacteria have mechanisms that allow them to break down phosphonates and/or phosphoesters when SRP is not readily available. Alkaline phosphatase (APA) is a broadly used enzyme for releasing phosphate from a phosphoester. Three bacterial APAs that are functionally equivalent but differ with respect to their cofactors are widely reported from oceanic waters. The most studied APA is PhoA, a homodimer that is activated by two Zn^2+^ ions and one Mg^2+^ ion (Roy et al., 1982; Sebastian and Ammerman, 2009). However, PhoA appears to be relatively low in abundance in the marine environment compared to other bacterial APAs: PhoX and PhoD. Structurally unrelated to PhoA, PhoX and PhoD are monomers and are activated by three Ca^2+^ and two Fe^3+^ ions (PhoX) or one Ca^2+^ and one Fe^3+^ (PhoD) (Majumdar et al., 2005; Rodriguez et al., 2014). PhoX and PhoD have been detected in multiple ocean taxa, including Cyanobacteria and Alpha- and Gamma-proteobacteria (Luo et al., 2009; Sebastian and Ammerman, 2009). More *phoD* sequences were found in the open ocean than *phoX* and *phoA* combined in a study of the APAs within the Global Ocean Sampling metagenomic database (Luo et al., 2009). Within that database, sequences were predominantly from free-living bacterioplankton. To our knowledge, neither P limitation nor APA activity have been reported in the bacteria associated with marine copepods.

Recent research has described bacterial communities associated with marine copepods (De Corte et al., 2014; Shoemaker and Moisander, 2015, 2017; Skovgaard et al., 2015; Datta et al., 2018), but relatively few studies have examined bacterial activities in the copepod microbiome (Scavotto et al., 2015; Almada and Tarrant, 2016; De Corte et al., 2018; Moisander et al., 2018). Here, we describe an exploratory metatranscriptomic profile of bacteria associated with open ocean copepods, with a specific emphasis on bacterial APAs. qPCR and RT-qPCR were used to further assess the potential for APA activity as a fitness strategy for copepod-associated bacteria.

## Materials and Methods

### Sample Collection

Copepods were collected from zooplankton net tows conducted in August 2013 and 2014 onboard the *R/V Atlantic Explorer*, as described elsewhere (Moisander et al., 2018; Shoemaker et al., 2019). Briefly, multiple net tows were conducted near BATS (31°50′ N 64°10′ W) between 16:30 and 5:20 local time. A 200 μm -mesh net with a sealed cod-end was towed at 50–90 m with minimal wire speeds (∼7 m min^–1^). Tow contents were immediately diluted with seawater and kept gently aerated. Individual living copepods were picked, rinsed in 0.2 μm filtered seawater (FSW), and sorted based on visual identification.

**FIGURE 1:**
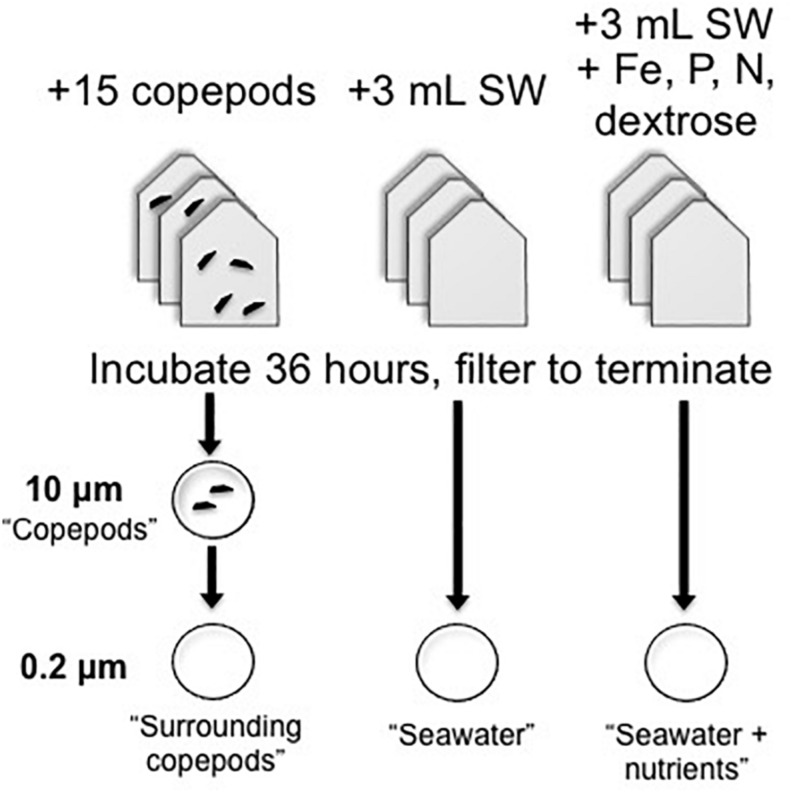
Experimental design of copepod and seawater incubations used in qPCR experiments. Triplicate experimental bottles were used for each treatment. For seawater treatments, 3 mL of whole seawater inoculum was added to 72 mL of 0.2 μm-filtered seawater. Copepods were added to 75 mL of 0.2 μm-filtered seawater. After incubation, samples were filtered onto 0.2 μm filters for seawater or serially through 10 and then 0.2 μm filters for the copepod-containing samples. Note that for the metatranscriptome samples, copepods and the surrounding bacterial community were collected directly on 0.2 μm filters.

Copepods were kept in bottle incubations to examine gene expression in the copepod-associated bacteria (Figure 1). The incubation treatments of bottles with picked copepods (∼15 per vial) were compared with treatments with seawater (SW) only or SW with a combination of nutrients that had final concentrations as follows: ${\text{NO}}_{3}^{-}$ (0.5 μM), ${\text{NH}}_{4}^{+}$ (0.5 μM), FeCl (0.5 μM) chelated with EDTA (0.5 μM), ${\text{PO}}_{4}^{3-}$ (1 μM), and a commonly bioavailable carbon source, dextrose (0.5 μM). The SW + nutrients treatment was expected to have lower expression of APA genes than the seawater treatment without nutrients, due to the addition of phosphate. Other potentially limiting or co-limiting nutrients were added to create a nutrient-replete environment. When a sufficient number of living copepods were collected, they were evenly dispersed based on copepod genus into each of the copepod incubation bottles containing FSW from the station at which copepods were collected. The experimental copepods included *Pleuromamma, Undinula*, and *Sapphirina*, among others. In most experiments multiple copepod genera were used, but even numbers and types of individuals were distributed into incubation vials. Gas-tight, glass serum bottles (75 mL) or polycarbonate bottles (125 mL) with 75 mL seawater were incubated for 36 h in the dark as described in Table 1. Additional SW was collected with Niskin bottles attached to a CTD-rosette immediately preceding the night-time tows and preserved for comparisons as ‘non-incubated samples.’ DNA was collected on 0.2 μm membrane filters (Supor, Pall Gelman) from 10, 200, 300, and 500 m from BATS in 2013 and from 20, 40, 80, 120, 200, 300, and 500 m from BATS in 2014. For analysis of these non-incubated samples, samples collected at 80 m and above were considered “Surface” samples, and those collected below the deep chlorophyll maximum (DCM; ≥ 120 m) were considered “Deep” samples.

**TABLE 1:** Experimental design in bottle incubations.

Exp. #	Zooplankton collection date	Copepod # and type per bottle	Depth of SW inoculum	Incubation volume (mL)
1	August 21, 2014	15 Mixed	10 m	125
2	August 22, 2014	15 *Undinula*	80 m	75
3	August 23, 2014	15 Mixed	40 m	125
Metatranscriptome	August 7, 2013	∼12 Mixed	N.A.	75
Metatranscriptome	August 8, 2013	10 *Undinula*	N.A.	75

*All samples were incubated for 36 h in the dark. Duplicate or triplicate samples were collected for each treatment within each experiment. N.A. = SW inoculum was not included.*

Samples from two experiments were analyzed for metatranscriptomes (in 2013; Table 1), and from three experiments using qPCR/RT-qPCR (in 2014). After 36 h, incubations were terminated by filtration. For metatranscriptomics, samples were filtered directly onto 0.2 μm membrane filters. Samples used for qPCR and RT-qPCR were first filtered through 10 μm polycarbonate filters to collect copepods, followed by 0.2 μm membrane filters to collect the 0.2–10 μm fraction. Filters were stored in separate autoclaved bead beater tubes containing ∼0.1 g each of 0.1 and 0.5 mm sterile glass beads, and the RNA samples were stored with 350 μl of RLT buffer (from the Qiagen RNeasy Mini Kit; Valencia, CA, United States) amended with β-mercaptoethanol. The RNA tubes were then frozen in liquid N. DNA samples were frozen at −80°C. Samples were transported to the laboratory in a liquid N dry shipper.

RNA was extracted from both 0.2 and 10 μm filters. Samples were first agitated for 2 min at maximum speed in a Mini-BeadBeater-8 (Biospec Products, Bartlesville, OK, United States), iced for 2 min, then agitated again. The samples were then centrifuged for 2 min at 8,000 *g*, and the filters were removed. The rest of the protocol followed the Qiagen RNeasy minikit protocol, followed by a TURBO DNase treatment (Invitrogen, Carlsbad, CA, United States). DNA was extracted from all experimental seawater and copepod samples using a modified DNeasy Mini Plant Kit (Qiagen) as previously described (Shoemaker and Moisander, 2015).

### Preparation and Analysis of Metatranscriptome

Metatranscriptomic sequencing was conducted to explore prokaryotic gene expression in copepod association. Six incubated copepod samples were prepared for metatranscriptome sequencing from two incubation experiments conducted in August of 2013 (Table 1). Each sample consisted of 10 copepods (either mixed genera or *Undinula*) and the bacterial community surrounding them captured on the 0.2 μm filter at the end of a 36 h incubation. Metatranscriptome samples were not size-fractionated to separate the copepods from the surrounding community; therefore, the reported gene expression is from both the copepod-attached bacteria and the bacteria in the copepod incubation water (the copepod “zoosphere”; Shoemaker et al., 2019).

RNA was extracted and purified with the Qiagen RNeasy Kit and then concentrated by overnight ethanol precipitation at −20°C. The pellet was washed twice in 70% ethanol, air dried, and eluted in 16 μl of RNase-free water. Ribosomal RNA was removed using the Microb*Express* (Life Technologies, Beverly, MA, United States) and Terminator Exonuclease enzyme (Epicentre, Madison, WI, United States). Poly-A tails were added with the Epicentre Poly(A) Polymerase Tailing Kit. Complementary DNA (cDNA) was synthesized and purified with the MessageAmp II Kit (Life Technologies, Beverly, MA, United States). A sequencing library was prepared with the Nextera XT DNA kit (Illumina, San Diego, CA, United States). One ng of sample was submitted for sequencing on the MiSeq platform at Tufts University Core Facility for Genomics (Boston, MA, United States).

Unpaired reads were imported to CLC Genomics Workbench 8.0 (Qiagen Bioinformatics, Redwood City, CA, United States) and were initially trimmed to only include sequences with quality higher than Q20, zero ambiguities, and reads longer than 50 bp. The Illumina adaptors were trimmed from the sequences, and poly A/T tails longer than 8 bp removed. Fasta files were processed in MG-RAST for pairing, further quality control, and analysis, using default settings. The sequences from all six samples were then pooled for further analysis (see section “Results”). Sequences were classified via MG-RAST using NCBI RefSeq for taxonomic identifications and both the KEGG Orthology and SEED subsystems databases for functional gene analysis. Sequences have been deposited in the NCBI Sequence Read Archive (accession SRP089826).

### Alkaline Phosphatase Gene Primer Design

The metatranscriptome data showed expression of genes related to APA gene transcription; these sequences were most closely related to Gammaproteobacterial *phoD* and *phoX*. We created clone libraries to examine the diversity of these genes within Gammaproteobacteria. Previously published PCR primers were used to amplify *phoX* genes (Sebastian and Ammerman, 2009), and *phoD* primers were designed by analyzing Gammaproteobacterial genomes available in Joint Genome Institute’s Integrated Microbial Genomes (JGI IMG). Representative clone libraries were created by ligating PCR products into the pGEM-T (Promega, Madison, WI, United States), and cloning in *E. coli* JM109. The clones were Sanger sequenced at the Massachusetts General Hospital DNA core facility (Cambridge, MA, United States). Sequences of *phoD* had closest blastn matches with *Alteromonas* spp. and *Vibrio* spp., while sequences from *phoX* had the highest identity with two *Vibrio* spp. GenBank accession numbers for the *phoD* and *phoX* sequences from this study are MN698650–MN698681. Four sets of qPCR primers were then designed using the NCBI Primer-BLAST (Ye et al., 2012) to separately quantify *phoD* in *Alteromonas* and *Vibrio*, and *phoX* in two phylotypes of *Vibrio* (Table 2).

**TABLE 2:** Primer sets for PCR and qPCR.

Name	Target	Length	Primers	qPCR efficiency
*phoD*	Gammaproteobacteria	795 bp	F: 5′-TWCAYCTYGGTGAYTACATTTATGARTA-3′ R: 5′-TCRACRKRGTARCCATCCCAWGCRTC-3′	
phoX*	Gammaproteobacteria	586 bp	F: 5′-GGGNACTTAYYTMACBTGYGAA-3′ R: 5′-GDCKATCCATBGKBGTTGC-3′	
*phoD*1	*Alteromonas* spp.	98 bp	F: 5′-TATAYATGCTCGACACCCGC-3′ R: 5′-AAAGCGYGCTTGGTCAAACG-3′	95%
*phoD*2	*Vibrio* spp.	103 bp	F: 5′-CGGTTTAGTTGCYCAGTCGC-3′ R: 5′-ATTCCAAGTTGCGTCGTGCG-3′	82%
*phoX*1	Vibrio splendidus**	91 bp	F: 5′-GAAGCGAAATGGGACCCACG-3′ R: 5′-TCGCCACATATAGMGTGCCT-3′	104%
*phoX*2	*Vibrio alginolyticus*	189 bp	F: 5′-GCTTGGCGATGGGTGACAAG-3′ R: 5′-CAACTAAATCCGCCGCACCC-3′	101%

The reverse primers of the Gammaproteobacterial *phoD* and *phoX* primers were used for cDNA synthesis. *From Sebastian and Ammerman (2009). All other primers were designed for this study. **Primer set *phoX*1 is expected to amplify other *Vibrio* spp. including *V. crassostreae* and *V. chagasii*; however, most of the *phoX*1 clones and metatranscriptome reads had high identity to *V. splendidus*.

qPCR standards were created using linearized plasmid DNA, purified with the GeneJet PCR kit (Thermo Fisher, Waltham, MA, United States). Plasmid DNA was quantified with PicoGreen (Invitrogen, Carlsbad, CA, United States) and plasmid concentration calculated based on the length of insert sequence and the plasmid size. Standards were diluted with nuclease free water (NFW) to 10^9^ gene copies (gc) μl^–1^ and working aliquots were made to 10^8^ gc μl^–1^ from the 10^9^ stock and stored at −20°C. New serial dilutions from 10^0^ to 10^7^ were made for each run of the 10^8^ stock with NFW.

### cDNA Synthesis for qPCR

cDNA was synthesized from RNA extracted from experimental samples using a standard protocol. Briefly, 4 μl of RNA was added to 0.5 μl of 10 mM dNTPs and 0.5 μl of the original reverse PCR primer (10 μM, *phoD* or *phoX*) in PCR tubes. The mixtures were incubated at 65°C for 5 min, placed on ice for 1 min, then combined with 5 μl of cDNA master mix (SuperScript III reverse transcriptase kit; Invitrogen, Carlsbad, CA, United States). For every sample, both the reverse transcriptase (RT) reaction and no-RT reaction NFW control was processed to check for residual DNA. The RT reactions were incubated at 50°C for 50 min, then 5 min at 85°C. Samples were returned to ice and 0.9 μl of RNaseH was added. Samples were incubated at 37°C for 20 min, then stored at −20°C.

### Quantitative PCR

Optimal primer concentration for the SYBR Green qPCR was first determined for each primer set by evaluating efficiency and melt curves of standards at final primer concentrations of 50, 125, and 250 nM. The optimal primer concentration was 50 nM for *phoD*2, and 125 nM for *phoD*1, *phoX*1, and *phoX*2. 18.4 μl of the master mix containing the primers with SYBR Select (Applied Biosystems, Foster City, CA, United States) was added to each well of a 96-well optical plate. 1.6 μl of either standard, cDNA, no-RT cDNA, or NFW [no template control (NTC)] was added as a template. Standards, RT reactions, and NTCs were run in technical duplicates (experimental triplicates) while no-RT reactions were analyzed as technical singletons (experimental triplicates). None of the NTCs or no-RT reactions amplified. All qPCR amplification reactions were completed on a StepOne Plus Real-Time PCR instrument (Applied Biosystems, Foster City, CA, United States). All reactions consisted of an initial denaturation step at 95°C for 10 min followed by 40 cycles of 95°C for 30 s and 60°C for 1 min. Melt curves were produced at the end of every run. Before analyzing data, melt curves were visually inspected for the presence of more than a single peak in melting temperature, to assess amplification specificity. A few samples amplified with *phoX*2 primers had a non-specific peak in the melt curve, and these samples were not included in analysis. No secondary peaks were observed in the melt curves produced with the other primer sets.

Gene and transcript abundances in the incubation bottles were calculated from Ct values and standard curves. The threshold for quantification was set to 500 gene copies per extract and the limit of detection was determined to be 62.5 gene copies per extract, corresponding to 8 and 1 gene copies per reaction, respectively. A conservative value of 60 was used for values falling between 62.5 and 500 gene copies per extract. A ratio of transcript abundance to gene abundance was calculated to normalize transcript abundance within the same treatment. A conservative estimate of 1 copepod per liter of seawater in the North Atlantic Ocean (Munk et al., 2010) was used when comparing data from copepods and seawater. Statistical analyses were done using IBM SPSS Statistics (v25). Data normality and homogeneity of variance were tested with the Shapiro–Wilks and Levine’s tests, respectively. Non-normal data were log transformed and re-tested. Normally distributed data were tested with One-way ANOVAs, and the non-parametric Mann–Whitney *U*-test was done if normality was not met. Box plots were made in R Studio with ggPlot2 (Wickham, 2016).

## Results and Discussion

### General Metatranscriptome Description

**FIGURE 2:**
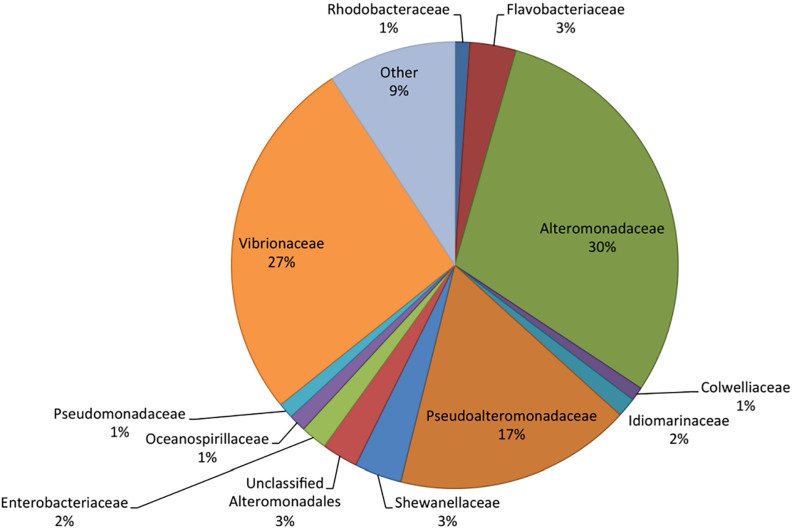
Taxonomic composition (RefSeq) of the bacteria in the metatranscriptome based on relative abundance of reads. RefSeq taxonomies were assigned to all functional genes and ribosomal RNA to determine community composition.

Metatranscriptome sequencing resulted in a total of 21,955,296 reads. Sequences were analyzed in MG-RAST (Meyer et al., 2008), resulting in 2,045,589 predicted features among the pooled samples. Of these, 77% were ribosomal RNA, while 18% were annotated proteins and 5% were unknown proteins. RefSeq analysis grouped 40% of the sequences as bacterial, 60% eukaryotic, and less than 0.2% as viral or archaeal. The majority of identified bacterial transcripts were identified as Gammaproteobacteria based on RefSeq, with 30% of the sequences identified as Alteromonadaceae, 27% as Vibrionaceae, and 17% as Pseudoalteromonadaceae (Figure 2). Flavobacteriaceae and Rhodobacteraceae made up 3 and 1% of the total bacterial community, respectively, which were lower proportions than previously reported in similar bottle incubations based on 16S rRNA gene amplicon sequencing of DNA (Shoemaker et al., 2019). The lower relative abundance of transcripts from these groups may indicate a bias in RNA extraction, an underrepresentation in the RefSeq database, or that other bacterial groups contained fewer transcripts per cell when compared to the Gammaproteobacteria.

All non-ribosomal bacterial reads from the six samples were pooled for functional analysis due to the relatively low coverage in the sequencing and the overall similar functional groupings observed within each sample (Supplementary Figure 1). All 28 of the non-ribosomal SEED Subsystem functional classifications from MG-RAST were present (Table 3 and Supplementary Figure 2). The most abundant sequences were from SEED subsystems for protein metabolisms (17.58%), clustering-based subsystems (ribosomes, proteosomes, and functional coupling; 14.29%), and carbohydrate metabolisms (9.45%). These commonly form the highest proportion of reads in studies of other marine environments, and it appears the sequencing effort gave an expected gene expression profile (Frias-Lopez et al., 2008; Gilbert et al., 2008). Pathways for cellular respiration and DNA replication were nearly complete. We expect that the community was actively growing and DNA replication was taking place, given the 36-h incubation time and the cell abundance measured in similar experiments (Shoemaker et al., 2019). The near completeness of these necessary cellular pathways indicates that the sequencing depth was in fact reasonably high after pooling the samples. We note that the aim of the metatranscriptome analysis was to explore any potential, highly expressed bacterial pathways in this understudied environment, and was not designed to provide data for statistical comparisons of samples. Additionally, these data reflect the metabolic potential of bacteria in copepod microbiomes and surroundings within a confined volume (Table 1). The higher-than-natural copepod density in the incubations may have influenced the results. This study serves as a novel exploration of copepod-associated bacterial metabolic potential; however, future gene expression studies should be conducted with natural populations and under more dilute incubation densities.

**TABLE 3:** SEED Subsystems classification and relative abundance of bacterial transcripts as classified within MG-RAST.

Subsystems classification	Transcript abundance	Percentage
Protein metabolism	38096	17.58
Clustering-based subsystems	30959	14.29
Carbohydrates	20470	9.45
Amino acids and derivatives	12453	5.75
Miscellaneous	12115	5.59
Stress response	11476	5.30
Membrane transport	11407	5.26
RNA metabolism	10826	5.00
Respiration	9367	4.32
Cell wall and capsule	7470	3.45
Cofactors, vitamins, prosthetic groups, pigments	6761	3.12
Motility and chemotaxis	6525	3.01
Nucleosides and nucleotides	5820	2.69
DNA metabolism	5409	2.50
Virulence, disease, and defense	4876	2.25
Fatty acids, lipids, and isoprenoids	4717	2.18
Iron acquisition and metabolism	3402	1.57
Regulation and cell signaling	2786	1.29
Cell division and cell cycle	2681	1.24
Phosphorus metabolism	2270	1.05
Phages, prophages, transposable elements, plasmids	1617	0.75
Nitrogen metabolism	1356	0.63
Potassium metabolism	1173	0.54
Metabolism of aromatic compounds	1085	0.50
Sulfur metabolism	837	0.39
Dormancy and sporulation	571	0.26
Secondary metabolism	140	0.06
Photosynthesis	28	0.01
**Total**	216,693	100

### Chemotaxis, Quorum Sensing, Pathogenicity, and Nutrient Pathways

The pathway for bacterial chemotaxis was complete, with over 5000 transcripts (2.3% of the total metatranscriptome reads) associated with chemotaxis present. Many of these transcripts had highest identity with the Gammaproteobacteria that often dominate copepod microbiomes collected from the area: (*Vibrio* (15%), *Pseudoalteromonas* (29%), *Alteromonas* (32%); Moisander et al., 2018; Shoemaker et al., 2019). A few additional bacterial groups were included (*Marinobacter*, *Shewanella*, *Saccharophagus*, and others). Marine bacteria that depend on transient nutrient patches benefit from chemotactic responses, as these responses allow them to reach the patch 10 times faster than bacteria that happen upon the patches by chance (Stocker et al., 2008). This ability to sense and move toward a copepod may be crucial for the establishment of the copepod microbiome and zoosphere (the latter referring to the bacterial communities immediately surrounding the copepod; Shoemaker et al., 2019).

Transcripts with homology to the LuxR-LuxI bacterial quorum sensing systems were identified. The transcripts were predominantly from *Vibrio* spp., however some transcripts were recovered that are classified as part of the *Pseudoalteromonas* LuxR family transcriptional regulators. LuxR homologs have been reported in quorum sensing marine *Pseudoalteromonas* spp. previously (Guo et al., 2011; Dang et al., 2017), but it is unknown if the bacteria in this study have an active quorum sensing system. At the DNA level, *Pseudoalteromonas* were present at very high numbers in similar incubations in another study, making up 16% of total sequences in a 16S rRNA amplicon sequencing analysis (Shoemaker et al., 2019). Quorum sensing regulates a variety of activities in bacteria, including biofilm formation, conjugation, and antibiotic production (Miller and Bassler, 2001). Quorum sensing activities could aid in the formation of biofilms on the copepod, which may ultimately help the bacteria stay attached while the host undergoes vertical migration and predator avoidance. Additionally, the bacteria could use quorum sensing to signal production of secondary metabolites and thereby reduce colonization of competing microbes. Antimicrobial activities mediated by quorum sensing have previously been reported in a sponge-associated *Pseudoalteromonas* sp. (Guo et al., 2011).

The metatranscriptome showed evidence for dissimilatory nitrate reduction to nitrite in Gammaproteobacteria associated with copepods, which was confirmed to be an active process in a parallel study (Moisander et al., 2018). Evidence for dinitrogen (N_2_) fixation, dissimilatory nitrate reduction to ammonium, or complete denitrification was not detected in the metatranscriptome. Some transcripts were present that could indicate the presence of sulfur and methane pathways (0.39 and 0.70% of the total transcripts, respectively). The pathway forming formate from formaldehyde was present and could indicate the presence of aerobic methane oxidation. Few transcripts were present for sulfite oxidation and the assimilatory sulfate reduction pathway was complete. Many of these genes have multiple uses within a cell, and we note that presence of these transcripts does not necessarily indicate use for a specific pathway.

### Phosphorus Pathways

Genes required for the activation and usage of APA and phosphonates were expressed. Within the metatranscriptome, 0.06% of all coding reads were for *phoH*, which encodes a phosphate starvation inducible protein; the majority of these sequences had a high identity to *Vibrio* spp. and *Pseudoalteromonas* spp. Additionally, 0.08% of the total transcripts encoded the phosphate regulon transcriptional regulator, PhoB, with high identity to the same genera. Activation of *phoX* is dependent on the PhoB regulatory protein (Zaheer et al., 2009). In culture conditions, *phoX* was only activated under P starvation (Sebastian and Ammerman, 2009). Under P starvation, pho regulation under PhoU is upregulated (Hirota et al., 2010). Transcripts identified as *phoU* were present and mostly had high identity to *Vibrio* and *Pseudoalteromonas.* The presence of these transcripts in the metatranscriptome suggests the bacteria associated with the copepods may be P starved.

The expression of APA genes indicates DOP utilization within the copepod zoosphere was likely driven by bacterial APA activity. 0.16% of the total coding reads were identified as APA transcripts, representing three known bacterial APA genes: *phoA, phoD*, and *phoX*. Of these, 48% had high identities with *Alteromonas*, 17% with *Vibrio*, and 7.3% with *Pseudoalteromonas.* The remaining APA transcripts mostly represented other Gammaproteobacteria and 4.1% of transcripts had the greatest identity with *Synechococcus*. Although *phoD* and *phoX* have been reported in a variety of bacteria in the open ocean (Luo et al., 2009; Sebastian and Ammerman, 2009), the APA transcripts in this study were almost entirely from Gammaproteobacteria. Yet, it is unlikely that only the Gammaproteobacteria in the copepod zoosphere experience P limitation. APAs may be produced and released extracellularly and the resulting phosphate shared with neighboring bacteria. Of the oceanic APA genes sequenced in the Global Ocean Survey, 30% were identified as coding for extracellular enzymes (Luo et al., 2009). Living in dense populations could benefit bacteria that are unable to produce their own APAs, and APA activity has previously been reported higher in particle-attached bacteria than in free-living bacteria (Hoppe and Ullrich, 1999; Labry et al., 2016; Davis and Mahaffey, 2017). In these studies, particle-associated bacteria produced high levels of APAs, even in SRP replete systems. One hypothesis is that APAs are produced when bacteria are in close contact with particles containing high levels of phosphoesters, even when P is not limited (Labry et al., 2016). Additionally, bacteria may benefit from the organic C released from breaking the C-O-P bond (Van Wambeke et al., 2002; Nicholson et al., 2006). It is possible that the copepod-associated bacteria in this study were not P limited; however, the surface waters of the oligotrophic North Atlantic are believed to be P-limited throughout the summer (Cavender-Bares et al., 2001). Therefore, copepod food sources are likely P-limited as well, which could affect nutrient availability within the copepod zoosphere.

Phosphonate utilization is another important mechanism bacteria use to access P in the open ocean (Clark et al., 1998; Kolowith et al., 2001). Only a single transcript of *phnJ* encoding for potential phophonatase activity was present in the metatranscriptome; thus, phosphonate utilization does not appear to play an active of role within the copepod zoosphere. Copepods release dissolved inorganic phosphorus (DIP) and DOP, although the type of DOP released is not known and is likely variable (Hargrave and Geen, 1968; Valdés et al., 2018). The ratio of released phosphoesters to phosphonates likely changes based on copepod diet. The expression of APA genes in the copepod-associated bacteria in this study indicates the DOP pools associated with the copepods were likely rich in phosphoesters.

### Alkaline Phosphatase Gene and Transcript Abundance in Incubation Experiments

The abundance of APA genes and their transcripts was quantified in seawater (SW) and copepod incubation samples from 2014 (Experiments 1–3; E1–E3), and in samples collected in 2013 and 2014 not subjected to incubation. Gene and transcript abundances associated with copepods and the water surrounding the copepods (copepod incubation water) were compared to gene and transcript abundances from SW treatments incubated in the absence of copepods. We expected to see elevated abundance and expression of APA in copepod incubations compared to SW treatments.

**FIGURE 3:**
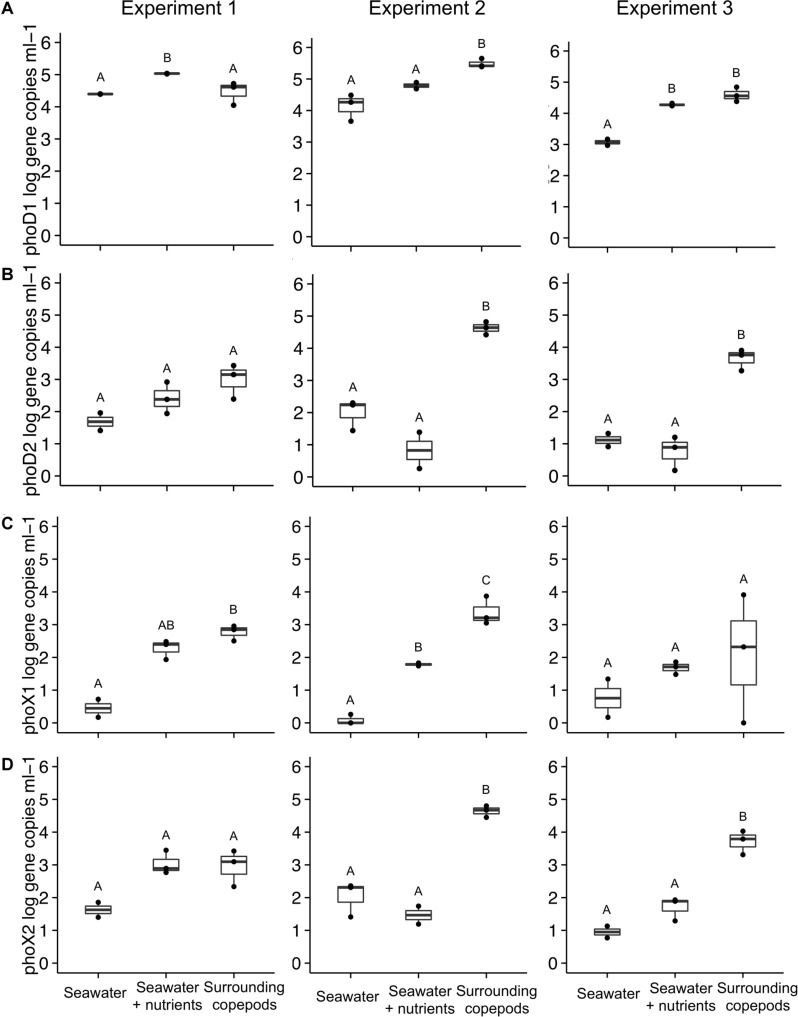
Alkaline phosphatase gene abundance in the three experiments. Four primer sets were used to amplify: **(A)** *phoD*1 (*Alteromonas* sp.), **(B)** *phoD*2 (*Vibrio* sp.), **(C)** *phoX*1 (*Vibrio* sp.), and **(D)** *phoX*2 (*Vibrio* sp.). Number of experimental replicates is shown with dots and was either *n* = 2 or *n* = 3. Whiskers are the first and third quartiles, with the mean shown as a solid line. Values are shown as log scale, although statistical testing was done with non-transformed data if data were normally distributed. All data are based on samples collected at the end of the incubation. The treatment “Surrounding copepods” refers to the 0.2–10 μm size fraction remaining after the collection of copepods on a 10 μm filter. Letters indicate significant differences between treatments based on ANOVA (*p* < 0.05).

In E1, *phoD*1 gene copies (*Alteromonas* spp.) were elevated more in the SW + nutrients than in the SW without nutrients, or in the water from the copepod incubations (ANOVA, *p* = 0.002, Figure 3A). In E2, however, *phoD*1 gene copy abundance in the water surrounding the copepods was higher than that in the SW treatments (ANOVA, *p* = 0.008). In E3, *phoD*1 gene abundance was elevated in SW + nutrients and in copepod incubation water, compared to SW without nutrients. The second *phoD* phylotype tested, referred to here as *phoD*2, was specific for *Vibrio* spp. Gene abundance of *phoD*2 was not significantly different between treatments in E1, but in E2 and E3, the water surrounding the copepods had higher *phoD*2 gene abundance than either of the SW treatments (ANOVA, *p* = 0.001 for both, Figure 3B). The primers designed to target *phoX* (*phoX*1 and *phoX*2) were specific to two phylotypes of *Vibrio* spp. (Table 2). In E2, the copepod incubation water had significantly higher *phoX*1 gene abundance than either SW treatment (ANOVA, *p* < 0.001, respectively). In E1, SW without nutrients had lower *phoX*1 count than the other treatments, while in E3 there were no significant differences (Figures 3A,C). For *phoX*2, in E2 and E3, gene abundance was significantly higher in the water surrounding the copepods than in the SW treatments (ANOVA, *p* < 0.001, ANOVA, *p* = 0.001, respectively); however, in E1 there was no difference between the water surrounding copepods and the SW + nutrients treatment (Figure 3D).

**FIGURE 4:**
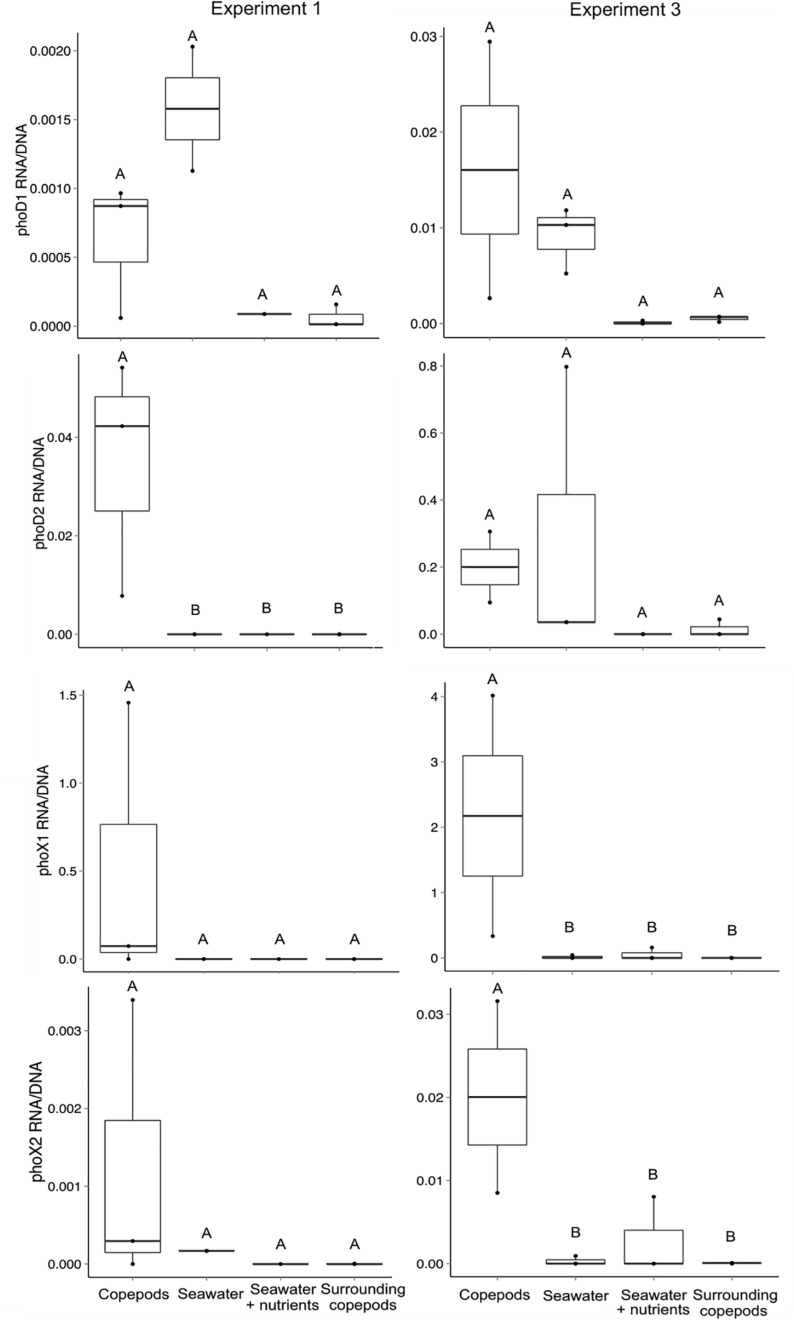
Transcript abundance normalized to gene abundance for the four alkaline phosphatase gene targets in experiments 1 and 3. The ratio of transcripts (copies of cDNA) to gene copies was calculated for all seawater treatments and the copepod size fraction. The treatment “Surrounding copepods” refers to the 0.2–10 μm size fraction remaining after the collection of copepods on a 10 μm filter. The number of experimental replicates is shown as dots and was either *n* = 2 or *n* = 3. Whiskers are the first and third quartiles, with the mean shown as a solid line. Letters indicate significant differences between treatments based on ANOVA (*p* < 0.05).

Relative levels of transcript abundance to gene abundance were determined for E1 and E3 for each of the four primer sets. While *Alteromonas phoD*1 transcripts were detected both in the presence and absence of copepods, the *Vibrio*-associated *phoD*2, *phoX*1 and *phoX*2 often had higher transcript per gene ratio in the presence of copepods. In E1, *phoD*2 had a higher ratio of expression to gene abundance in the copepod size fraction than in any other treatment (Mann–Whitney, *p* = 0.024, Figure 4). The increased expression of *phoD*2 in association with copepods suggests that the *Vibrio* APA is expressed when the bacterium is attached to the host. The comparatively lower expression in the water surrounding the copepods indicates this activity is specific to the copepod host. These results support the idea that these *Vibrio* spp. have adapted to a P limited environment on open ocean copepods.

In E3, both *phoX*1 and *phoX*2 had significantly higher expression on the copepod size fraction than in the water surrounding the copepods or in the SW treatments (Mann–Whitney, *p* = 0.036 for both, Figure 4). The differences between experiments may be explained by slight variation in incubation conditions, including the collection depth for the incubation water (10, 80, and 40 m for E1, E2, and E3 respectively). Although these experiments were done in a closed system, the presence and expression of Gammaproteobacterial APA genes indicates that APA utilization is likely an important pathway by which copepod-associated bacteria, specifically those in physical contact with copepods, can access P and C in the North Atlantic Ocean.

### Abundance of Alkaline Phosphatase Genes in Non-incubated Copepods and Seawater

**FIGURE 5:**
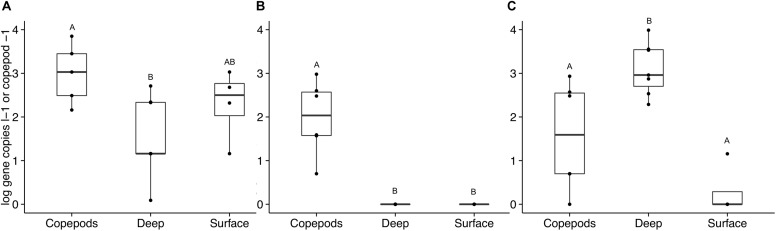
Alkaline phosphatase gene abundance in copepods and seawater samples collected and preserved directly without incubation. Bulk seawater measurements (“Surface” and “Deep”) were taken from the surface to 500 m in 2013 and 2014. The “Surface” seawater grouping includes samples from 10 to 80 m (above the DCM, *n* = 4). The “Deep” seawater grouping includes samples from 120 to 500 m (*n* = 6). The copepods were pooled into groups of 15 copepods (*n* = 6). Gene abundances are presented on a log scale as either per copepod or per liter of seawater. Letters indicate significant differences between treatments based on ANOVA (*p* < 0.05). Three primer sets were used to amplify these non-incubated samples: **(A)** *phoD*1, **(B)** *phoD*2, and **(C)** *phoX*2. No amplification was detected with the *phoX*1 primer set for these samples.

The occurrence of APA genes was investigated in samples collected directly from the water column to assess the overall ecological importance of the findings. DNA from seawater from the surface to 500 m collected in 2013 and 2014 was analyzed for the presence of the *phoD* and *phoX* targets. The *phoD*1 gene was present in the water samples with an average of 250 gene copies l^–1^ (*SD* = ± 327). There was no significant difference between water samples collected from depths above and below the deep chlorophyll maximum (DCM). The copepods directly preserved from net tows had a significantly higher number of *phoD*1 genes per copepod than per liter of seawater from below the DCM (ANOVA, *p* = 0.008). *phoD*2 was not detected in the SW at any depth; however, it was present on the copepods (mean = 288 gene copies copepod^–1^, *SD* = ± 364, ANOVA, *p* < 0.001, Figure 5).

*phoX*1 was present in only a few non-incubated samples at abundances so low that they were considered negligible in both the SW and copepod samples. In the non-incubated samples, *phoX*2 was detected primarily in the SW below the DCM and in the copepods. SW samples from below the DCM had significantly higher numbers of *phoX*2 genes than SW samples collected from above the DCM (means = 2.7 × 10^3^ ± 3.4 × 10^3^ and 3.0 ± 7.0 gene copies l^–1^ respectively, ANOVA, *p* < 0.001, Figure 5). Some copepods in the Sargasso Sea vertically migrate 100s of meters at night, and at depth they are surrounded by nutrient concentrations much greater than what is available to free-living bacteria near the surface (Steinberg et al., 2000). The SRP concentration at BATS increases below the DCM, while the DOP pool stays relatively constant (Lomas et al., 2010). Therefore, the large number of free-living *Vibrio* that contain *phoX* found below the DCM should experience a lessened need for APA in these deep layers. Bacterial hitchhiking (Grossart et al., 2010) on the copepods could potentially bring these *Vibrio* to the surface where there is a greater need to express APA. The disparity in gene abundance between incubated and non-incubated samples is likely due to the transient nature of bacterial attachment on copepods. The bottle incubations were designed to capture the bacterial communities growing on and being released from copepods over a period of time (36 h). Bacterial abundances higher than in ambient seawater were recorded previously for these experiments (Shoemaker et al., 2019). Movement of copepods in the water column would be expected to disperse and seed bacteria to the surrounding waters, while diluting the bacterial communities attached to and surrounding the copepods.

Overall, the primary bacterial group identified as transcriptionally active in the copepod zoosphere was Gammaproteobacteria. The data presented here indicate that bacteria actively seek the copepod environment via chemotaxis and use quorum sensing strategies in these environments. Presence and transcription of Gammaproteobacterial APAs suggests that bacteria sustained within the microzones surrounding copepods in the North Atlantic Gyre may be inorganic P-limited relative to the availability of other nutrients. In these oligotrophic waters, the copepod-associated Gammaproteobacteria appear to overcome P- and/or possibly C- limitation by hydrolysis of organophosphoesters, presumably originating from the copepods. The APA activity by select copepod-associated bacteria may produce a shared P and/or C resource to the surrounding community, regardless of nutrient limitation. More extensive enzyme activity and gene expression studies are needed to study activities of these pathways in the copepod association. Measurements conducted while minimizing experimental manipulation will be specifically informative. There appears to be rapid and taxon-specific microbial recycling of inorganic and organic matter originating from copepods in oligotrophic waters. The biogeochemical processes taking place within copepod microenvironments are important to consider given the high numerical abundances and wide geographic distribution of copepods in the ocean.

## Data Availability Statement

The datasets generated for this study can be found in the NCBI Sequence Read Archive: SRP089826, GenBank: MN698650–MN698681.

## Author Contributions

PM, EM, and KS designed the study and conducted the experiments. EM collected the metatranscriptome data. KS and EM analyzed the data. KS designed the primers and collected the alkaline phosphatase data. KS and PM wrote the manuscript.

## Conflict of Interest

The authors declare that the research was conducted in the absence of any commercial or financial relationships that could be construed as a potential conflict of interest.
