# Supplementary material for: Copepod-Associated Gammaproteobacterial Alkaline Phosphatases in the North Atlantic Subtropical Gyre
Source: Front Microbiol. 2020 May 25;11:1033. doi: 10.3389/fmicb.2020.01033 (PMC7261903; doi:10.3389/fmicb.2020.01033)
Supplement: Supplementary file 1 [file Data_Sheet_1.PDF]

Supplementary Figures

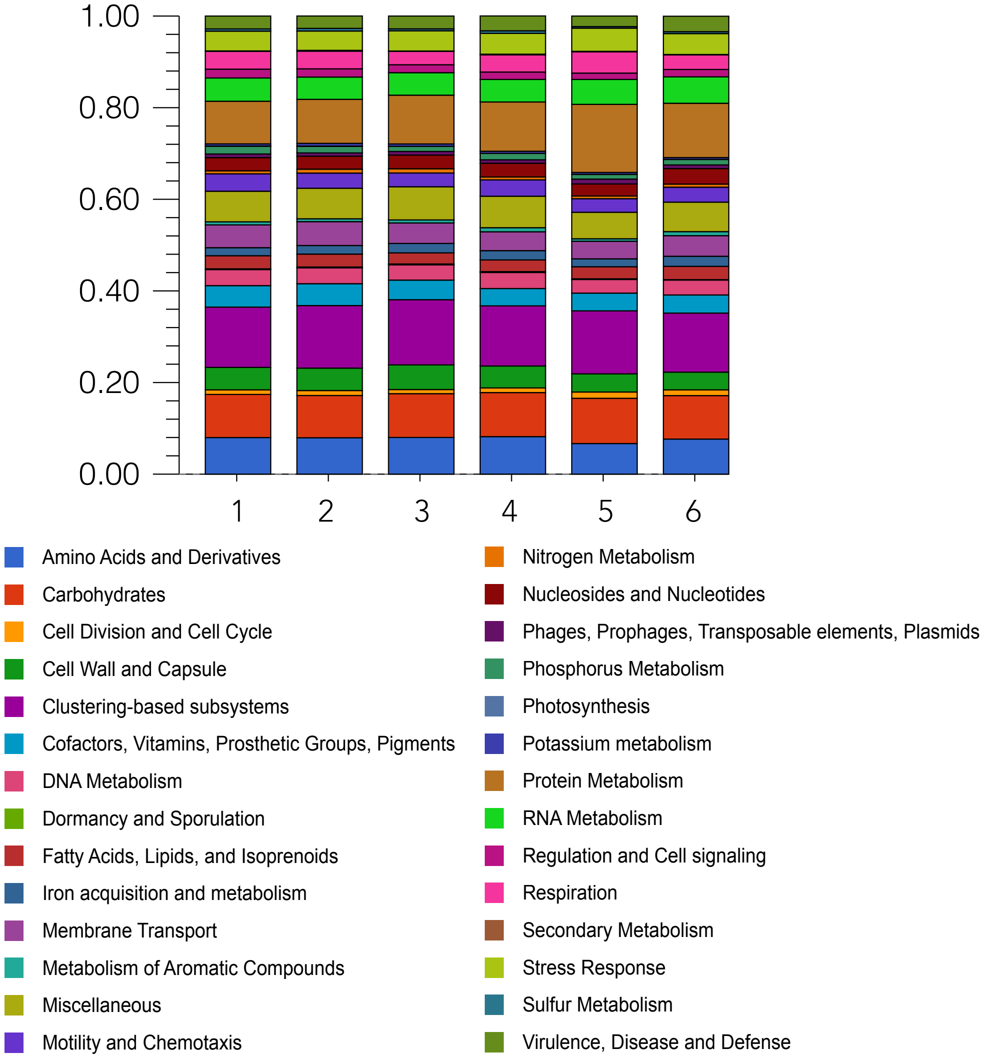

Supplementary Figure 1. MG-RAST subsystem clustering in the six metatranscriptome samples. The six experimental copepod incubation samples were nearly identical in functional gene expression at the subsystem level and were pooled for further analyses.

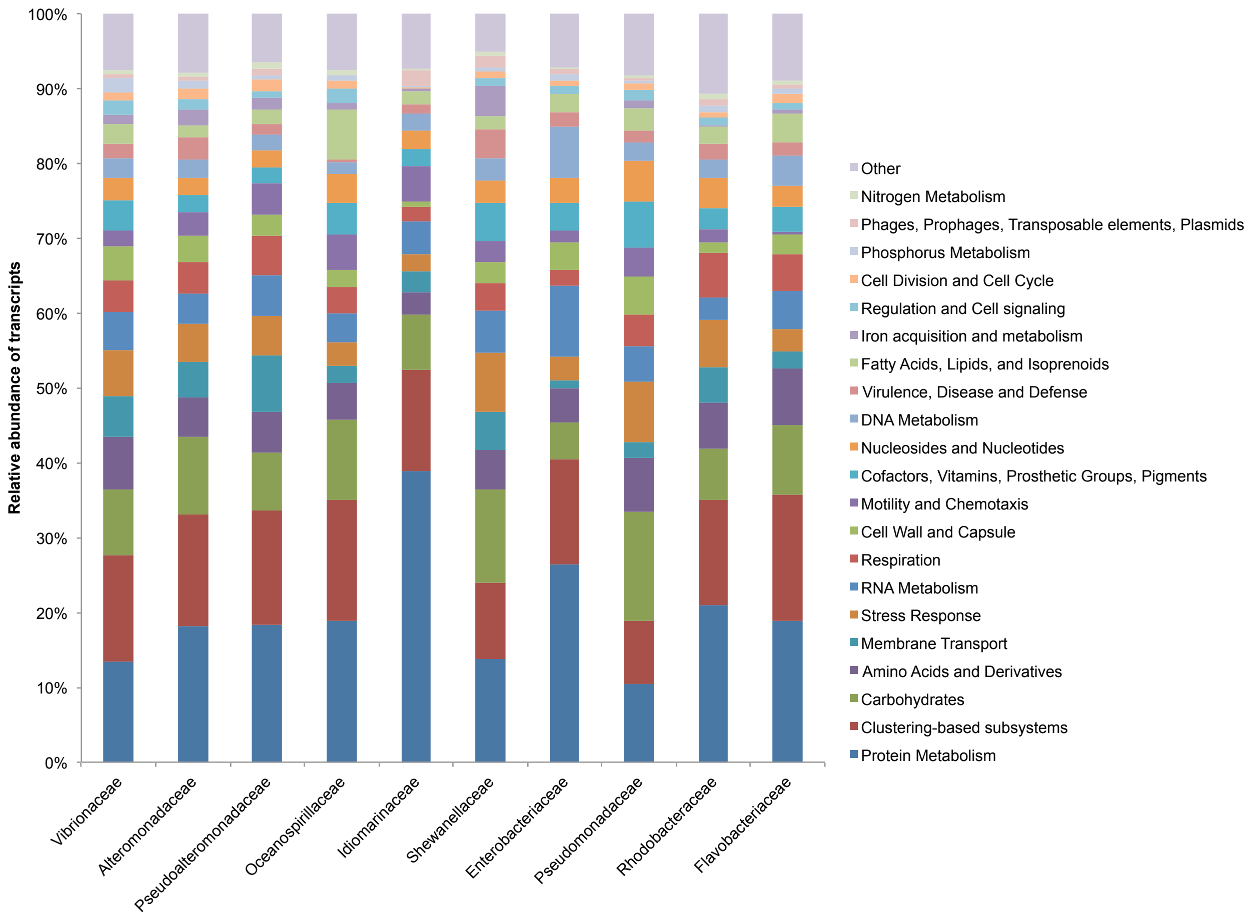

Supplementary Figure 2. Taxa-specific subsystems composition. Transcripts were assigned to a family and subsystem within MG-RAST. Gammaproteobacterial families with the highest number of transcripts are shown along with Rhodobacteraceae and Flavobacteriaceae.
